# New Appliances and Things Medical

**Published:** 1900-01-06

**Authors:** 


### NEW APPLIANCES AND THINGS MEDICAL.

[We shall be glad to receive, at our Office, 28 & 29, Southampton Street, Strand, London, W.C., from the manufacturers, specimens of all new preparations and appliances which may be brought out from time to time.]

### MALTZYME AND MALTZYME WITH COD LIVER OIL.

(MALT DIASTASE COMPANY, NEW YORK. AGENT: SYDNEY WERTIMER, 10, CROSS STREET, HATTON GARDEN, LONDON, E.C.)

MALTZYME appears a reliable and stable preparation containing a large percentage of the active ferment of malt; it has the advantage over most malt preparations in being clearer, thinner, and more limpid, while its malting properties are greater. It contains a certain proportion of phosphates and digested protein and starch products, consequently it is a food as well as a digestive and therapeutic agent. Those who find difficulty in digesting farinaceous foods, and yet require them for the maintenance of nutrition, will find maltzyme an excellent preparation. It has also been skilfully combined with cod liver oil, and this preparation should be substituted for the simple maltzyme where the therapeutic effects of cod liver oil appear to be additionally indicated.

### SCRUBB'S CLOUDY AMMONIA AND ANTISEPTIC SOAP.

(SCRUBB AND CO., GUILDFORD STREET, LAMBETH, LONDON, S.E.)

THIS valuable preparation of refined ammonia is a most advantageous substitute for soda. Not only does it remove grease and other stains from articles of clothing, furniture, and general household goods, but for use in the bath and other toilet purposes it is most efficacious as a cleansing medium. For the hair it is exceedingly invigorating, imparting a most desirable lustre. In cleaning the hands it is particularly useful, as it does not cause the same roughness of the skin as follows the application of soda. Many authorities, recognising the value of ammonia as a substitute for soda for the removal of dirt and grease from the skin, since being exceedingly volatile it immediately disappears after completing its duties, instead of remaining fixed in the pores of the integument, as is the case with soda or potash, have approached Messrs. Scrubb and Co. with the view that they should prepare a soap which, being free from the above objections as regards the presence of free potash or soda, might be used in conjunction with their cloudy ammonia, and combine at once the advantages of a soap and those of a cloudy ammonia. The result is the appearance of Scrubb's Antiseptic Skin Soap, a pure emollient preparation with no alkaline properties. Combined with Scrubb's Ammonia it will be found exceedingly valuable for the preservation of a soft and delicate skin; especially is this the case where the water is naturally hard.

### DEVONSHIRE CIDER.—"WHIMPLE'S SPECIALITE" BRAND.

(HY. WHITEWAY AND CO., WHIMPLE, DEVON.)

WE have received samples of this most excellent brand of bottled cider. It is a peculiarly delicious aud exhilarating beverage, possessing withal a low percentage of alcohol. Pure, carefully manufactured cider of this kind is a particularly wholesome beverage for the gouty and rheumatic, and, unlike malted liquors, it has no fattening properties. Indeed, some authorities regard the fermented juice of the apple as particularly applicable to the cases of those who suffer from an excess of adipose tissue. The chief virtues, however, of this particular brand are its purity and its fineness of flavour.

